# Outpatient depression current care expenditure changes in Liaoning Province from 2015 to 2020: a study based on the “system of health accounts 2011”

**DOI:** 10.3389/fphar.2024.1092580

**Published:** 2024-01-22

**Authors:** Yuedan Ma, Xiaoxia Shi, Kristin K. Sznajder, Yue Zhao, Quan Wan, Peipei Chai, Xiaoshi Yang

**Affiliations:** ^1^ Department of Traditional Chinese Medicine, School of Graduate Students, Liaoning University of Traditional Chinese Medicine, Shenyang, China; ^2^ Department of Public Health, School of Medicine, Pennsylvania State University, Hershey, PA, United States; ^3^ China National Health Development Research Center, Beijing, China; ^4^ Department of Social Medicine, School of Health Management, China Medical University, Shenyang, China

**Keywords:** depression, outpatient expenditure, CCE, SHA 2011, burden of disease

## Abstract

**Introduction:** Depression is the leading cause of disability worldwide and has become a health issue of global concern. Based on the “System of Health Accounts 2011” (SHA 2011) for patients with depression, this paper studies the changes in the current curative expenditure (CCE) of outpatient depression in Liaoning Province, China, and provides policy recommendations.

**Method:** A stratified multistage random sample of 56,994 patients with depression included from 1,227 healthcare facilities in Liaoning Province were included. The significance of differences in variables within groups was analyzed by univariate analysis (including descriptive statistics analysis, Mann-Whitney U test and Kruskal–Wallis H test), and factors influencing depression outpatient CCE were analyzed by multiple linear regression analysis and constructing structural equation models (SEM).

**Results:** The CCE of outpatient depression was ranging from CNY 75.57 million to CNY 100.53 million in 2015–2020, with the highest of CNY 100.53 million in 2018, CNY 103.28 million in 2019. Medical expenditures are mainly concentrated in general hospitals and provincial healthcare institutions, accounting for about 90% of all provincial scope expenditures. The multiple regression results show that provincial healthcare institutions, purchase of drug, select medical treatment for depression, general hospitals and urban employees’ health insurance are the main influencing factors for depression outpatient CCE. The results of SEM show that insurance status negative impact outpatient expenditure.

**Conclusion:** Health insurance is an important factor in equitable access to healthcare resources for patients, and medication expenditure is the influential factor affecting the high expenditure of outpatient clinics. It is of great importance to reduce the medical burden of patients by increasing the coverage of medical insurance, increasing the proportion of bills that are eligible for reimbursement, and improving the system by guaranteeing the supply of psychotropic medication.

## 1 Introduction

Depression has four indicators of alarm: high prevalence, high relapse rate, high disability rate and high suicide rate. Depression has been a leading cause of disability globally for decades (Collaborators, 2018), accounting for 1.8% of global disability-adjusted life years (DALYs) and is the second leading cause of death after cancer (Wu et al., 2010). According to the World Health Organization (Lepine and Briley, 2011; Organization, 2012), 280 million people globally suffered from depression in 2019, and depression is the leading causative factor for mental illness in the next decade. Depression affects approximately 3.8% of the global population (Institute for Health Metrics and Evaluation, 2019), with prevalence rates ranging from 3.9% to 6.0% (2.63–5.45 million people) in European countries (including the United Kingdom, Russia, Germany, Italy, and Spain), 4.9% (15.29 million people) in the United States, and 2.7% (3.26 million people) in Japan (Institute for Health Metrics and Evaluation, 2019). In the UK, 1.7 billion, £203.5 billion is spent annually on depression treatment, medication purchases (National Institute for Clinical Excellence, 2009; Health and Social Care Information Centre, 2017). Globally, depression and anxiety disorders cost the world up to $1 trillion per year (World Health Organization, 2017). In addition to causing death, depression reduces worker productivity and increases the risk of absenteeism. Even with aggressive cognitive-behavioral therapy and medication, all depressive symptoms are rarely eliminated, and the risk of persistence and relapse is high. As China’s economy grows, the number of people with depression has gradually increased, reaching 5,005.5 million in 2019, accounting for 3.7% of China’s total population (Institute for Health Metrics and Evaluation, 2019), and is one of the main contributors to the number of disability-adjusted life years in China in 2010 (Yang et al., 2013). The prevalence of depression and DALYs are higher in northeastern China (Ren et al., 2020). Due to the high disability and prevalence of depression, depression is mainly treated through medication and counseling, which is a long and costly treatment cycle. As a result, costs associated with the treatment of depression are likely to account for a significant proportion of healthcare costs.

One study used econometric modeling to examine healthcare costs associated with depression and depressive symptoms, estimating projected healthcare costs for depression and depressive disorders based on a two-step approach with a two-part model and four-part model coefficients (Hsieh and Qin, 2018) and analyzing antidepressant use and expenditures based on evidence from urban claims data in China (Ding et al., 2022). Patient and self-medication visits (Jin et al., 2022), as well as the economic burden of hypertension and depression (Wu et al., 2021). Were assessed based on data from the China Health and Retirement Longitudinal Study (CHARLS) database and using a zero-inflated Poisson regression model, including a Logit model predicting multiple zeros and a Poisson count model. Previous studies have analyzed and estimated healthcare expenditures for depression in China based on information from different databases, but it is likely that each study used a different estimation model, which is less informative for assessing overall expenditures for depression in China. Currently, a study in Shandong Province analyzed hospitalization costs for childhood depression (Guo et al., 2019), but to our knowledge, no studies have been found that provide a systematic accounting framework for subnational depression costs, and the lack of analysis of the costs of different dimensions, such as the extent and type of depression healthcare institutions, financing structure, and disease type, reduces the accuracy of the measurements and the different countries’ Comparability.

There is a need to determine how to estimate the economic burden of depression in a highly accurate and recognized way. The current highly recognized and widely used methodology for measuring healthcare costs is SHA2011. SHA2011 was revised by the Organization for Economic Cooperation and Development, Eurostat (EUROSTAT), and the World Health Organization, which jointly organized a group of experts on health costing to follow the System of National Accounts (SNA) and the Principles of Health Cost Accounting (PNA). This led to the establishment of an international health costing classification and statistical reporting system that reflects the sources of health financing. The separate allocation and use of health funds makes health costs measured using this system more relevant, feasible and sustainable.

Therefore, based on the SHA2011 accounting framework and using sample data from Liaoning Province, this study measured the outpatient costs of depression in Liaoning Province and further assessed the relationship between outpatient services in terms of healthcare institutions, degree and type of disease, financing structure, beneficiary populations, and economic burden of depression, which can lead to a more meaningful analysis of healthcare resources, cost containment, and control strategies, and provide policy recommendations for the province of Liaoning, and even for China Provide policy recommendations for depression cost control.

## 2 Materials and methods

### 2.1 Data sources

Four sources contributed to the research data. The Statistical Yearbook of Health and Family Planning of Liaoning Province (2015–2020), the Liaoning Provincial Health Financial Annual Report (2015–2020), the Liaoning Provincial Government Health Investment Monitoring Data (2015–2020), and the Liaoning Provincial Statistical Yearbook, are the statistical data from the health and related administrative departments that represent the total cost of treatment services in Liaoning Province (2015–2020). Sample data from the multistage stratified probabilities-proportional random sampling approach was used to collect patient healthcare costs, population data and clinical information about healthcare facilities (Ma et al., 2020; Fang et al., 2021). Dalian, Fushun, Jinzhou, Panjin and Tieling cities were selected to conduct random, multistage spot checks in the first analytic phase, including the following variables GDP, national income, national income *per capita* and population density. In the second phase, the quality of data collection and the accuracy of health information systems (medical insurance system, patient consultation information record system, and medical and health cost details) were included from one district and two counties were selected in each sample city totaling 15 district and county sample sites identified. In the third phase, the medical and health institutions in the 15 sample districts and counties were selected according to the type and level of medical institutions, including 83 public health institutions (disease prevention and control institutions, maternal and child health institutions, health education institutions, emergency centers, blood centers, family planning guidance institutions, health supervision institutions and specialized disease control institutions), 83 medical institutions (public hospitals and private hospitals), 1061 grassroots institutions (community health service institutions, health centers, outpatient clinics, clinics, and village health offices), totaling 1,227 medical and health institutions. Through the health information system, the medical records of outpatients and inpatients from various institutions were gathered and a database was created with data on gender, age, region, date of consultation, initial diagnosis, disease name, type of medical institution, total cost, cost details and insurance type (Zhai et al., 2015). The sample data were cleaned and checked using the ICD-10 disease classification code to determine the severity of diseases. Only incident depression diagnoses were chosen for the sample data and additional diagnoses were not taken into account. To find, recode or delete ICD-10 data that does not adhere to the standard, we used Excel’s VLOOKUP function. We also cleaned and coded gender, age, cost, institution type, institution level and insurance type following the export template for outpatient and inpatient expenses of the health information system.

### 2.2 Study samples

The study sample included patients with depression who were initially diagnosed by a professional clinician at a healthcare facility between 1 January 2015 and 31 December 2020. The sample inclusion criteria used the International Classification of Diseases, 10th Revision (ICD-10) to identify F32-F33 as patients with depression. Patients with depression are classified as F32-F33; 1) mild depression (F32.0, F33.0); 2) moderate depression (F32.1, F33.1); 3) major depression (F32.2, F32.3, F33.2, F33.3); 4) other depression (F32.8, F32.9, F33.8, F33.9) (Ding et al., 2022). For all diseases we treated as missing values the disease name and disease ICD-10 of the outpatient visit information submitted by the sample organizations as null values, and we had to discard such data because we could not take estimation or other replacement supplementation methods for the null values of the disease name and disease ICD-10. Liaoning province based on SHA2011 accounting for the total cost of health mental illness specialized hospitals only in Dalian Seventh People’s Hospital, and part of the year in the sample pool does not have data for this institution, in view of the uniformity of the data sample institutions, so it was decided to remove the sample of depression in this institution. Among all health facilities, the cost of hospitalization was determined to be too low after screening the sample data, thus only the financial burden of outpatients diagnosed with depression was investigated (Table 1).

**TABLE 1:** Composition of outpatient and hospitalization expenditure in Liaoning Province, 2015–2020.

Year	Outpatient expenditure (million (%))	Hospitalization expenditure (million (%))	Total
2020	98.04 (95.93)	4.16 (4.07)	102.20 (100.00)
2019	103.28 (93.74)	6.89 (6.26)	110.17 (100.00)
2018	100.53 (92.26)	8.44 (7.74)	108.97 (100.00)
2017	97.86 (95.66)	4.43 (4.34)	102.29 (100.00)
2016	85.99 (93.94)	5.55 (6.06)	91.54 (100.00)
2015	75.57 (91.04)	7.44 (8.96)	83.01 (100.00)

### 2.3 Quality control and data management

Sample data were collected from the total health cost accounting management system by professional clinicians at all levels and in all types of medical institutions based on disease diagnoses recorded as ICD-10 codes. Relevant personnel involved in the study received professional training from experts at the National Health Commission’s Centre for Health Development Studies conducted an on-site evaluation of the effectiveness of the training, and only those who passed the assessment could participate in the formal data processing. During the data collection process, the missing, incorrect or unreasonable data provided by the agency in the total health cost accounting management system were identified, returned to the source agency, and resubmitted to the system upon change or addition. Patient personal information for the sample data was digitally coded, and names and specific identities were not disclosed, therefore the local institutional review board waived patient consent. Sample data collected were cleaned and screened according to ICD-10 disease classification codes, and only patients diagnosed with depression for the first time were selected to have multiple disorders in the sample data, regardless of other co-morbidities. The VLOOKUP function was used in Microsoft Excel (Excel 2013; Microsoft Corporation, Redmond, Washington, USA) to find, recode, or delete non-compliant ICD-10 data for gender, age, cost, facility type, facility classification and insurance types. Data were cleaned and coded according to the Health Information System outpatient and inpatient cost export templates. All processed sample data were statistically and statistically analyzed using STATA 15.0 (Stata Corp of Texas, USA).

### 2.4 Estimating CCE for depression in the frame of SHA 2011

SHA2011 is a standard technique for policy analysis and financial flow description that is similar globally. The distribution of beneficiaries, institutional allocation and the flow of health funding, including long-term care, rehabilitation and treatment services are all described in detail. The total outpatient revenue of various institutions at all levels was derived from the official statistical data of the Liaoning Provincial Health Statistical Yearbook and the Liaoning Provincial Financial Annual Report based on the SHA2011 theoretical framework. The Liaoning Province CCE was calculated using the outpatient diagnosis of depression based on the institution type (general hospital, traditional Chinese medicine hospitals, specialized hospitals, specialized public health institutions, maternal and child health institutions, primary medical institutions, outpatient departments), age group (5 years old as a gradient) and various disease categories. Outpatient CCE included income from outpatient treatment and subsidies for the basic expenses of outpatient treatment. Outpatient curative income was mainly the income obtained by medical institutions from the provision of routine medical services, the basic expenditure subsidy of outpatient clinics was the financial input subsidies provided by the government to guarantee the routine functioning of medical institutions. The formula is as follows:

$$S_{OCCE}=S_{OCI}+S_{OCBES}$$

In the above formula, $S_{\text{OCBES}}$ indicates outpatient CCE. $S_{\text{OCI}}$ indicates outpatient curative income, including treatment fee, drug fee, registration fee, consultation fee, check fee, surgery fee, test fee, and other fees. Outpatient curative basic expenditure subsidy included personnel expenses, and public expenses (office expenses, printing expenses, travel expenses, water and electricity expenses, postal and telecommunication expenses, vehicle expenses, special materials, conference expenses, training expenses, etc.). To calculate outpatient curative income, it is necessary to first obtain the total outpatient income of various types of medical institutions at all levels from the Liaoning Provincial Statistical Yearbook and the Liaoning Provincial Financial Annual Report, and then exclude the preventive service costs of various types of institutions at all levels in the case database of the sample institutions. The formula is:

$$S_{\text{OCI}}=S_{\text{TOI}}\times \left(1-\frac{\alpha_{p}}{\alpha }\right)$$

The above formula $S_{\text{TOI}}$ represents the total outpatient revenue of Liaoning Province, $\alpha_{p}$ represents the revenue from outpatient preventive services of representative sample institutions, α represents the total outpatient revenue of sample institutions, $\frac{\alpha_{p}}{\alpha }$ represents the proportion of preventive service revenue of representative sample institutions to total outpatient revenue, $1-\frac{\alpha_{p}}{\alpha }$ represents the proportion of outpatient treatment service revenue of representative sample institutions to total outpatient revenue.

The formula for calculating the actual outpatient treatment income represented by the cases in the sample institutions is as follows:

$$S_{\text{iOCI}}=S_{\text{OCI}}\times \frac{\alpha_{i}}{{\alpha -\alpha }_{p}}$$

In the above formula, $\alpha_{i}$ indicates the total EXP for cases in sample institutions, $\frac{\alpha_{i}}{{\alpha -\alpha }_{p}}$ represents the ratio of the total EXP of a case to the sum of the total EXP of all cases in the current sample institution, and $S_{\text{iOCI}}$ refers to the value of the actual outpatient treatment income that a case represents.

After calculating the real value of outpatient treatment income represented by cases, the income from outpatient treatment in different dimensions was obtained by adding the age groups and disease types. The age group was based on a gradient of 5 years old, and the disease type was based on four categories of the global burden of disease (GBD) and 22 categories of ICD_10 outpatient treatment income from different perspectives. The formula is as follows:

$$S_{\text{nOCI}}=\sum_{i=1}^{n}\left(S_{\text{OCI}}\times \frac{\alpha_{i}}{\alpha -\alpha_{p}}\right)$$

The outpatient curative basic expenditure subsidy was obtained from the financial basic appropriation income of various medical institutions at all levels from the Liaoning Provincial Statistical Yearbook and the Liaoning Provincial Financial Annual Report, as the total outpatient curative basic expenditure subsidy, and the service volume selects the general emergency of various medical institutions at all levels, including the number of visits and total hospital bed days. The basic expenditure subsidy was calculated based on workload and did not include the proportion of outpatient preventive services in the sample institutions as a proportion of the total number of consultations in the sample institutions. The formula is as follows:

$$N_{OCI}=N_{TOI}\times \left(1-\frac{N_{p}}{N_{OS}}\right)$$

In the above formula, $N_{TOI}$ represents total outpatient and emergency visits, $N_{OCI}$ represents the total number of treatment services, $N_{p}$ represents the number of outpatient preventive services in sampled institutions, $N_{OS}$ represents the total number of outpatient visits in sampled institutions, $\frac{N_{p}}{N}$ represents service outpatient as a percentage of the total number of visits to sampled institutions. Due to the difference between the workload of physicians’ outpatient clinics and inpatient workloads, the basic expenditure subsidy for therapeutic outpatient clinics was calculated according to the service volume, and the service volume was unified. The calculation formula of the proportion of hospitalized bed days to the total service volume was as follows:

$$P_{IS}=\frac{N_{IS}}{N_{IS}+N_{OCI}*K}$$

In the above formula, $P_{IS}$ represents the proportion of inpatient services to total services, $N_{IS}$ represents total hospital bed days, K is a constant according to the recommendation of the National Health Development Research Center of China, K=0.1 .

To calculate the outpatient curative basic expenditure subsidy, the proportion of inpatient services in the total service volume was excluded. The calculation formula is as follows:

$$S_{\text{OCBS}}=S_{\text{TOCBS}}\times \left(1-P_{\text{IS}}\right)$$

In the above formula, $S_{\text{TOCBS}}$ represents fiscal basic appropriation income obtained from statistical yearbooks and financial annual reports.

The formula of the actual outpatient treatment basic expenditure subsidy represented by the cases in the sample institutions is as follows:

$$S_{\text{iOBES}}=S_{\text{OCBES}}\times \frac{\alpha_{i}}{\alpha -\alpha_{p}}$$

In the above formula, $S_{\text{iOBES}}$ represents the case represents the actual basic expenditure subsidy for outpatient treatment.

After calculating the actual outpatient treatment basic expenditure subsidy represented by the case, the outpatient treatment basic expenditure subsidy of different dimensions was obtained according to the age group and the type of disease. The age group was based on a gradient of 5 years old, and the disease types are subsidized for basic outpatient treatment from different perspectives in four categories of GBD and 22 categories of ICD_10. The formula is as follows:

$$S_{\text{nOCBES}}=\sum_{i=1}^{n}\left(S_{\text{OCBES}}\times \frac{\alpha_{i}}{\alpha -\alpha_{p}}\right)$$

To calculate CCE through financing plans, government plans (basic expenditure subsidies, medical assistance for urban and rural residents), social medical insurance plans (urban workers, urban residents, new rural cooperative medical system, work injury, unemployment, pension, maternity insurance), commercial medical insurance, non-profit organization financing (charitable donations), corporate financing (medical assistance for corporate employees), and household personal hygiene expenditures include residents’ hygiene expenditures were included (Patel et al., 2007).

### 2.5 Factors influencing outpatient expenditures

We screened a total of 56,994 depression outpatient sample data from a total of 32,185,646 outpatient sample data over 6 years (5,726 outpatient patients with depression were gathered in 2015, followed by 6,462 in 2016, 5,066 in 2017, 12,220 in 2018, 10,910 in 2019 and 16,610 in 2020). Univariate analysis was used to set dummy variables for categorical variables for subsequent analysis, including descriptive statistics. Dichotomous variables included whether to purchase drug, whether to select treatment and sex, therefore, using Mann-Whitney U test yielded *p* < 0.05 as inclusion criteria for multifactorial analysis and multicategorical variables included age, insurance status, institution level, institution type and year, and the Kruskal–Wallis H test was used to derive *p* < 0.05 as inclusion criteria for multifactorial analysis. The factors influencing depression outpatient CCE were analyzed by using the logarithmically transformed depression outpatient costs as the dependent variable and including all independent variables that underwent univariate analysis by multiple linear regression analysis. We used IBM SPSS Statistics V.25.0 (IBM Corp) for univariate and multifactor analyses of depression outpatient clinics. AMOS Graphics, V24.0 (SPSS) was used to construct SEM to explore the factors influencing the cost of depression outpatient clinics.

### 2.6 Patient and public participation

Data for this study were obtained directly from the Total Health Cost Accounting System. Therefore without patient and public participation.

## 3 Results

### 3.1 The basic result of depression

Since 95% of depressed patients were concentrated in outpatient clinics, we studied only outpatient depressed patients with CCE. Generally speaking, from 2015–2020, the CCE of depression increased from CNY 75.57 million in 2015 to CNY 98.04 million in 2020, with the highest of CNY 100.53 million in 2018, CNY 103.28 million in 2019. In comparison to these 6 years of results, depression outpatient CCE 2015-2019 has been growing fast and then slow, with a slight drop in CCE in 2020, possibly due to the novel coronavirus affecting the normal operation of healthcare facilities, which has the same trend as CCE/GDP. However, the proportion of all disease CCE over 0.06%, was an unstable change trend. Depression treatment costs *per capita* also increased from CNY 1.72 in 2015 to CNY 2.30 in 2020 (Table 2).

**TABLE 2:** Distribution of outpatient CCE for depression in the province from 2015 to 2020.

Year	Depression outpatient CCE(million)	The proportion of all diseases CCE(%)	The proportion of GDP (%)	Depression CCE *per capita* (yuan)
2015	75.57	0.072	0.003	1.72
2016	85.99	0.070	0.004	1.96
2017	97.86	0.066	0.004	2.24
2018	100.53	0.067	0.004	2.31
2019	103.28	0.064	0.004	2.37
2020	98.04	0.061	0.004	2.30

### 3.2 Distribution of CCE among different groups

For the percentage of results of different subgroups of depression outpatient CCE, the main focus was on the choice to purchase medication, choice of treatment, female, 15-64 years, self-pay, provincial health facilities and general hospitals. The highest percentage of answering “yes” to whether to purchase drug was 97.32% in 2015. The highest value was 92.68 million CNY in 2019. The highest percentage of answering “yes”to whether to purchase drug was 67.72% in 2016, with a maximum value of 58.23 million CNY in 2016. The highest percentage of sex was 69.66% for females in 2020, with a maximum value of 68.30 million CNY. Age group share was highest at 87.39% in 2016 and maximum at 86.83 million in 2019. Insurance status share was highest at 88.02% self-pay in 2015 and maximum at 82.96 million CNY in 2020. Institutional level share was highest at 90.45% in 2017 for provincial health facilities and the maximum is 92.89 million CNY in 2019. The highest percentage of institution type is 96.93% for general hospitals in 2018 and the maximum is 97.44 million CNY in 2019 (Table 3).

**TABLE 3:** Distribution of outpatient expenses for depression by whether to purchase drug, whether to select treatment, sex, age, insurance status, institution level and type of medical institution, 2015–2020 (million (%)).

	2015	2016	2017	2018	2019	2020
**Whether to purchase drug**						
Yes	73.54 (97.32)	82.59 (96.05)	93.75 (95.80)	91.92 (91.43)	92.68 (89.74)	87.59 (89.34)
No	2.03 (2.68)	3.340 (3.95)	4.11 (4.20)	8.61 (8.57)	10.60 (10.26)	10.45 (10.66)
**Whether to select treatment**						
Yes	49.13 (65.01)	58.23 (67.72)	52.59 (53.74)	55.38 (55.08)	53.40 (51.70)	56.85 (57.98)
No	26.44 (34.99)	27.76 (32.28)	45.27 (46.26)	45.15 (44.92)	49.88 (48.29)	41.19 (42.02)
**Sex**						
Female	48.84 (64.62)	55.10 (64.08)	64.53 (65.94)	67.21 (66.86)	67.43 (65.29)	68.30 (69.66)
Male	26.73 (35.38)	30.89 (35.92)	33.33 (34.06)	33.32 (33.14)	35.85 (34.71)	29.74 (30.34)
**Age**						
0–14	0.33 (0.43)	0.24 (0.28)	0.63 (0.64)	1.72 (1.71)	3.75 (3.63)	5.07 (5.17)
15–64	64.37 (85.18)	75.14 (87.39)	83.89 (85.72)	86.27 (85.82)	86.83 (84.07)	81.90 (83.54)
≥65	10.87 (14.39)	10.60 (12.33)	13.34 (13.63)	12.54 (12.47)	12.71 (12.30)	11.07 (11.29)
**Insurance status**						
Urban employees’ basic medical insurance	8.43 (11.16)	10.81 (12.57)	13.94 (14.24)	13.58 (13.51)	14.97 (14.49)	14.84 (15.13)
Urban residents’ basic medical insurance	0.02 (0.03)	0.24 (0.28)	2.15 (2.20)	3.30 (3.29)	6.24 (6.04)	0.18 (0.18)
New rural cooperative medical care	0.09 (0.12)	0.14 (0.16)	0.18 (0.18)	0.32 (0.32)	0.59 (0.57)	0.07 (0.07)
Self-funded	66.52 (88.02)	74.57 (86.72)	81.45 (83.23)	82.54 (82.11)	81.48 (78.89)	82.96 (84.62)
**Institution level**						
Provincial level	67.45 (89.25)	77.68 (90.33)	88.52 (90.45)	90.29 (89.81)	92.89 (89.93)	87.62 (89.37)
Municipal level	8.03 (10.63)	8.22 (9.56)	8.92 (9.12)	10.02 (9.97)	10.26 (9.94)	9.88 (10.08)
District level	0.07 (0.09)	0.07 (0.08)	0.36 (0.37)	0.20 (0.20)	0.11 (0.10)	0.38 (0.39)
Country level	0.02 (0.03)	0.03 (0.03)	0.06 (0.07)	0.02 (0.02)	0.03 (0.03)	0.16 (0.16)
**Institution type**						
General hospital	71.36 (94.43)	79.29 (92.20)	92.83 (94.86)	97.44 (96.93)	97.55 (94.45)	89.78 (91.58)
Traditional Chinese medicine hospital	4.12 (5.45)	6.44 (7.49)	4.43 (4.53)	3.07 (3.05)	5.28 (5.11)	7.87 (8.03)
Specialized hospital	0.09 (0.11)	0.27 (0.31)	0.37 (0.38)	0.00 (0.00)	0.02 (0.02)	0.31 (0.31)
Primary medical institutions	0.00 (0.00)	0.00 (0.00)	0.08 (0.08)	0.00 (0.00)	0.40 (0.39)	0.02 (0.02)
Outpatient service agencies	0.00 (0.00)	0.00 (0.00)	0.14 (0.15)	0.02 (0.02)	0.05 (0.04)	0.05 (0.06)
**Total**	75.57 (100.00)	85.99 (100.00)	97.86 (100.00)	100.53 (100.00)	103.28 (100.00)	98.04 (100.00)

Bold font represent the collective term for the categories.

### 3.3 Allocation of CCE for different types of depression

Overall, the CCE for different types of depression showed a rising and then falling trend, the changing trend of CCE for other depression was consistent, for mild depression and major depression CCE kept increasing with the year, for moderate depression CCE showed a falling trend from 2015–2018 and gradually rebounded from 2018–2020. ICD-10 mainly divided depression into four categories, other depression CCE is the highest, followed by moderate depression, then by major and mild depression. In 2019, other depression outpatient treatment costs peaked at CNY 103.28 million. The highest cost for mild depression was CNY 3.10 million in 2020 and the lowest cost was CNY 0.74 million in 2015. The highest cost of moderate depression is CNY 5.07 million in 2020 and the lowest is CNY 4.33 million in 2018. The highest cost of major depression was CNY 2.52 million in 2020 and the lowest was CNY 0.59 million in 2015 (Table 4).

**TABLE 4:** CCE of different types of depression (CNY million).

Year	Total	Mild depression	Moderate depression	Major depression	Other depression
2015	75.57	0.74	4.45	0.59	69.80
2016	85.99	1.11	4.50	0.78	79.60
2017	97.86	1.83	4.57	1.02	90.44
2018	100.53	2.16	4.33	1.51	92.53
2019	103.28	2.55	4.56	2.03	94.14
2020	98.04	3.10	5.07	2.52	87.34

### 3.4 Distribution of CCE by age

**FIGURE 1:**
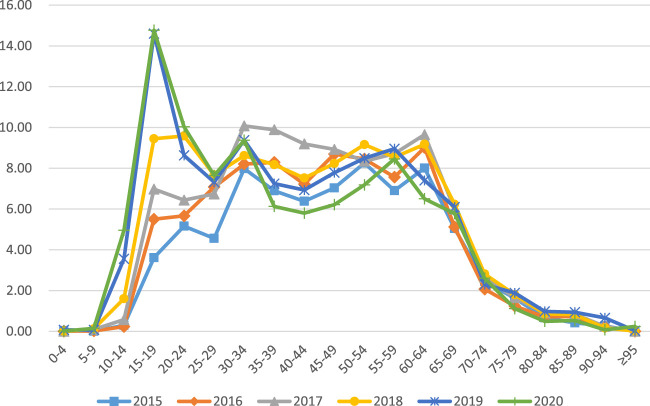
Age distribution of depression outpatients CCE 2015–2020(CNY million).

The CCE of depression varies greatly by age group. In general, CCE for depression starts to increase rapidly after the age of 14, peaking at CNY 14.77 million and CNY 14.60 million for the 15–19 age group in 2020 and 2019, peaking at CNY 9.58 million for the 20–24 age group in 2018, peaking at CNY 10.08 million in the 30–34 age group in 2017, the 60–64 age group peaked at CNY 9.01 million in 2016, the 50–54 age group peaked at CNY 8.25 million in 2015. The age group from 15–64 is dominant in the 2015–2020 fee and shows a wave shift for each age group. However, those below 15 years and above 64 years contribute less to depression CCE(Figure 1).

### 3.5 Health financing schemes

**FIGURE 2:**
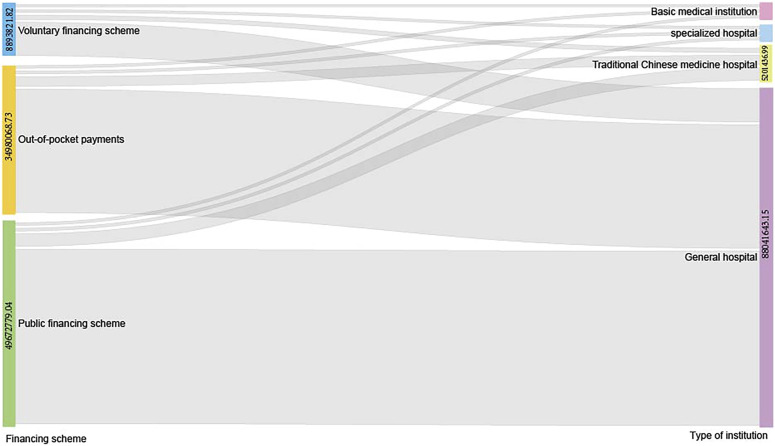
Sankey diagram for the average CCE in 2015–2020 associated with depression by financing schemes and type of institutions. CCE, current curative expenditure.

From 2015 to 2020, out-of-pocket (OOP) costs first increased by CNY 7.11 million, from CNY 30.01 million in 2015 to CNY 37.12 million in 2018, and then decreased by CNY 0.36 million, from CNY 37.12 million in 2018 to CNY 36.76 million in 2020. Meanwhile, the OOP share first decreases by 4.71%, from 39.72% in 2015 to 35.01% in 2019, then increases by 2.49% from 35.01% in 2019 to 37.50% in 2020. Between 2015 and 2020, public financing first increased by CNY 19.58 million, from CNY 38.18 million in 2015 to CNY 57.76 million in 2019, then decreased by CNY 4.77 million from CNY 57.76 million in 2019 to CNY 52.99 million in 2020. The public financing share shows an opposite trend to the OOP share, first increasing by 5.40%, from 50.52% in 2015 to 55.92% in 2019, then decreasing by 1.87% from 55.92% in 2019 to 54.05% in 2020. Overall, public financing and basic social health insurance are the main sources of funding for outpatient depression costs, with an overall OOP share of more than 35% and a smaller voluntary financing share of less than 10% (Table 5). The average cash flow from 2015 to 2020 from the “three services and one business” to four types of medical institutions: general hospitals, specialty hospitals, traditional Chinese medicine hospitals, primary healthcare institutions (community health centers, community health service stations, township health centers, health centers, village health centers, etc.) are shown in the Sankey diagram (Figure 2). The three financing schemes flow mainly to general hospitals, followed by traditional Chinese hospitals. General hospital financing is dominated by public financing and OOP.

**TABLE 5:** Distribution of financing expenses for depression clinics in Liaoning Province from 2015 to 2020 (million (%)).

year	Public financing scheme			Voluntary financing scheme			Out-of-pocket payments	Total
	Total	Government financing scheme	Social health insurance	Total	Social donation	Enterprise financing plan		
2015	38.18 (50.52)	6.66 (8.82)	31.52 (41.70)	7.38 (9.76)	1.17 (1.55)	6.21 (8.21)	30.01 (39.72)	75.57 (100.00)
2016	44.01 (51.18)	7.96 (9.26)	36.05 (41.92)	8.84 (10.28)	1.66 (1.93)	7.19 (8.36)	33.14 (38.53)	85.99 (100.00)
2017	51.50 (52.63)	9.47 (9.67)	42.04 (42.96)	9.67 (9.88)	2.69 (2.75)	6.97 (7.13)	36.69 (37.49)	97.86 (100.00)
2018	53.59 (53.30)	9.86 (9.81)	43.72 (43.49)	9.83 (9.78)	1.95 (1.94)	7.88 (7.84)	37.12 (36.92)	100.53 (100.00)
2019	57.76 (55.92)	10.80 (10.46)	46.96 (45.46)	9.36 (9.07)	1.66 (1.61)	7.71 (7.46)	36.16 (35.01)	103.28 (100.00)
2020	52.99 (54.05)	9.50 (9.69)	43.49 (44.36)	8.28 (8.45)	1.18 (1.20)	7.10 (7.24)	36.76 (37.50)	98.04 (100.00)

### 3.6 Factors influencing outpatient expenditures

Descriptive analysis, Mann-Whitney U test and Kruskal–Wallis H test of depression outpatient costs according to the number of independent samples included in different groups showed that depression outpatient costs were significantly different (*p* < 0.001) by whether to purchase drug, whether to select treatment, sex, age, insurance status, institution level, institution type and year. (Table 6).

**TABLE 6:** Differences in outpatient depression expenditure by subgroup (n = 56994).

Variables	n (%)	Outpatient expenditure Median (IQR)	Z/H	*p*-Value
**Whether to purchase drugs**				
Yes	44377 (77.90)	444.66 (213.73–791.7425)	−101.611^a^	<0.001
No	12617 (22.10)	76.40 (19.20–278.00)		
**Whether to select treatment**				
Yes	20807 (36.50)	499.70 (281.60–929.66)	−75.195^a^	<0.001
No	36187 (63.50)	272.16 (68.42–551.54)		
**Sex**				
Female	31897 (56.00)	340.81 (127.03–658.22)	−4.933^a^	<0.001
Male	25097 (44.00)	356.25 (138.43–707.00)		
**Age**				
0–14	1557 (2.70)	370.40 (188.02–671.52)	187.605^b^	<0.001
15–64	47668 (83.60)	357.64 (139.61–690.57)		
≥65	7769 (13.60)	295.80 (88.56–604.17)		
**Insurance status**				
Urban employees’ basic medical insurance	11272 (19.80)	347.92 (166.68–622.31)	191.906^b^	<0.001
Urban residents’ basic medical insurance	1369 (2.40)	276.80 (75.90–523.46)		
New rural cooperative medical care	267 (0.50)	125.20 (26.00–342.00)		
Self-funded	44086 (77.40)	354.00 (125.80–704.20)		
**Institution level**				
Provincial level	44911 (78.80)	405.80 (181.00–766.10)	4602.559^b^	<0.001
Municipal level	10644 (18.70)	221.31 (72.84–408.24)		
District level	637 (1.10)	23.69 (3.00–66.80)		
Country level	802 (1.40)	26.00 (5.53–164.46)		
**Institution type**			1462.719^b^	<0.001
General hospital	53271 (93.50)	345.16 (131–669.94)		
Traditional Chinese medicine hospital	3031 (5.30)	486.79 (236.2–1044.16)		
Specialized hospital	86 (0.20)	300.99 (58.0775–533.72)		
Primary medical institutions	500 (0.90)	19.59 (3–53)		
Outpatient service agencies	106 (0.20)	41.825 (30–165)		
**Year**			929.699^b^	<0.001
2015	5726 (10.00)	380.14 (182.71–695.29)		
2016	6462 (11.30)	427.40 (222.48–788.58)		
2017	5066 (8.90)	345.72 (163.00–630.00)		
2018	12220 (21.40)	303.90 (89.07–588.68)		
2019	10910 (19.10)	290.10 (79.21–598.32)		
2020	16610 (29.10)	378.57 (147.64–765.03)		

^a^ Stands for applying the Mann-Whitney U test (for 2 independent samples).

^b^ Stands for non-parametric Kruskal–Wallis H test (for k independent samples).

IQR, means percentile level.

Bold font represent the collective term for the categories.

The influencing factors of outpatient expenditure of depression in Liaoning Province were analyzed by multiple regression in Table 7. The included independent variables include whether to purchase drug, whether to select treatment, sex, age, insurance status, institution level, institution type and year. There were multiple covariates between the independent variables and no covariates between the independent variables and the response variables, so all independent variables were included in the regression equation (*p* < 0.01) and the linear model explained 38.4% of the variation in total outpatient costs. From the standardized regression coefficients, the positive effect on depression outpatient costs was in the order of provincial healthcare institutions, purchase drug, municipal level, select treatment, 15–64 age group, etc. The negative influence on the cost of depression outpatient clinics was in the order of traditional Chinese medicine hospital, general hospital, primary medical institutions, etc.

**TABLE 7:** Multiple regression analysis of impact factors on outpatient expenditure.

	Unstandardisation coefficient		Standardisation coefficient	T	Sig
	B (95%CI)	SE	Beta		
**Whether to purchase drug**					
Yes	0.697 (0.687–0.707)	0.005	0.453	133.799	<0.001
No	References				
**Whether to select treatment**					
Yes	0.361 (0.352–0.370)	0.005	0.272	76.031	<0.001
No	References				
**Sex**					
Female	−0.017 (-0.025–0.008)	0.004	−0.013	−3.726	<0.001
Male	References				
**Age**					
0–14	0.116 (0.088–0.144)	0.014	0.030	8.16	<0.001
15–64	0.061 (0.049–0.074)	0.006	0.035	9.747	<0.001
≥65	References				
**Insurance status**					
Urban employees’ basic medical insurance	0.061 (0.05–0.072)	0.006	0.038	11.013	<0.001
Urban residents’ basic medical insurance	0.113 (0.084–0.141)	0.015	0.027	7.737	<0.001
New rural cooperative medical care	0.007 (-0.057–0.07)	0.032	0.001	0.21	0.834
Self-funded	References				
**Institution level**					
Provincial level	0.860 (0.823–0.896)	0.019	0.550	45.605	<0.001
Municipal level	0.599 (0.561–0.637)	0.019	0.365	31.019	<0.001
District level	−0.098 (-0.171–0.025)	0.037	−0.016	−2.635	0.008
Country level	References				
**Institution type**					
General hospital	−0.696 (-0.813–0.579)	0.06	−0.269	−11.673	<0.001
Traditional Chinese medicine hospital	−0.768 (-0.887–0.65)	0.06	−0.270	−12.724	<0.001
Specialized hospital	−1.001 (-1.159–0.843)	0.081	−0.061	−12.417	<0.001
Primary medical institutions	−0.918 (-1.025–0.812)	0.054	−0.134	−16.961	<0.001
Outpatient service agencies	References				
**Year**					
2015	−0.127 (-0.142–0.111)	0.008	−0.060	−16.008	<0.001
2016	−0.059 (-0.075–0.044)	0.008	−0.029	−7.729	<0.001
2017	−0.036 (-0.052–0.020)	0.008	−0.016	−4.306	<0.001
2018	−0.141 (-0.154–0.129)	0.006	−0.091	−21.945	<0.001
2019	−0.115 (-0.128–0.102)	0.007	−0.071	−17.317	<0.001
2020	References				

Note: *R* ^2^ = 0.384,*p* < 0.001.

B, non-standardized regression coefficient. SE, Standard Error. Beta, standardization regression coefficient. *t*, *t*-test value (t-statistic). Sig, the meaning of coefficient (*P*).

95%CI, 95% Confidence Intervals.

Taking logarithms of outpatient expenditure in multiple regression analysis.

Bold font represent the collective term for the categories.

### 3.7 Modeling and model estimates

**FIGURE 3:**
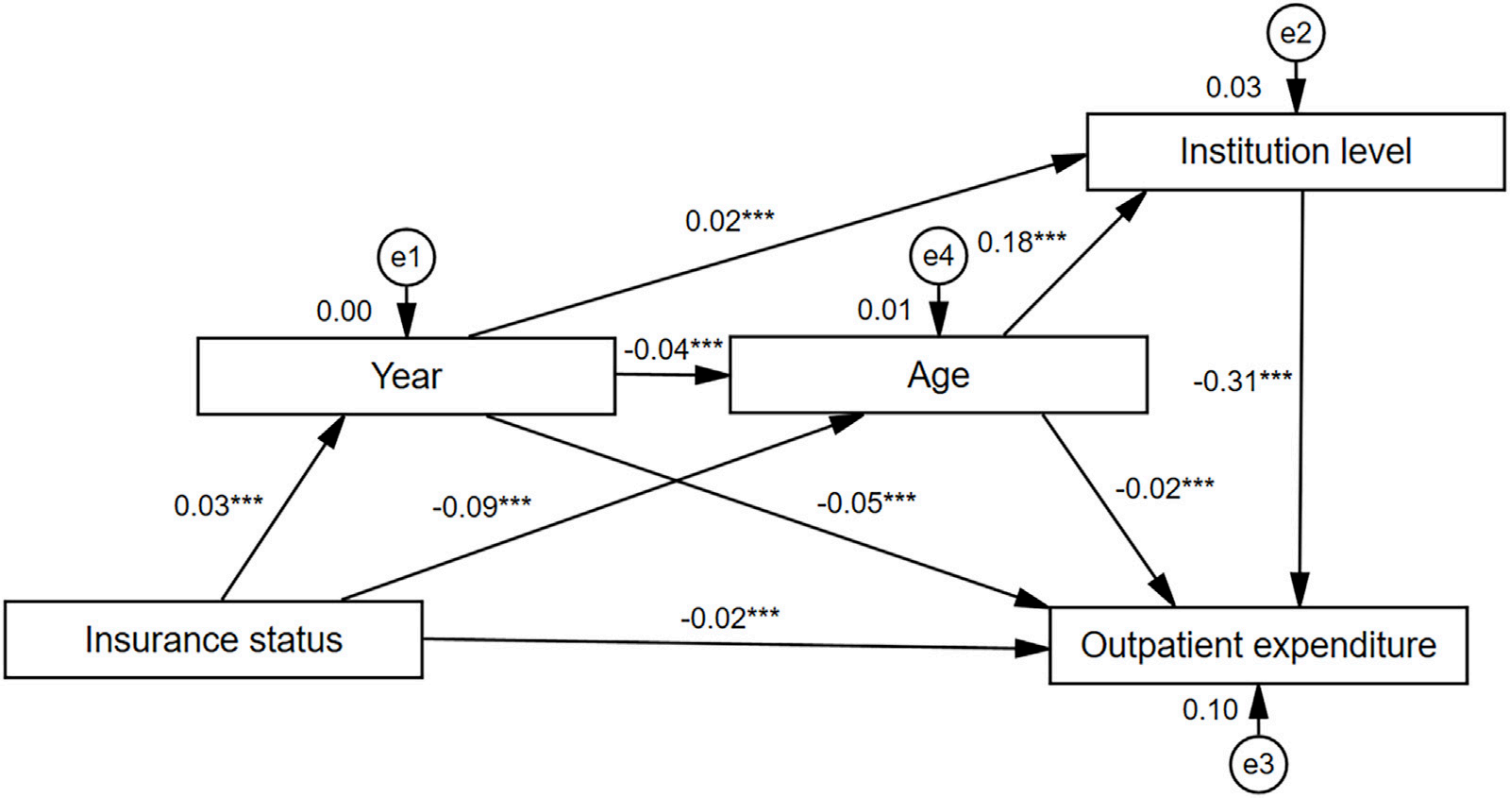
Path diagram of the structural equation model. Year contain 2015、2016、2017、2018、2019、2020. Age:0–14 = 1,15–64 = 2,≥65 = 3. Insurance status (in order of actual reimbursement ratio): Basic medical insurance for urban workers (actual reimbursement ratio = 75.60% (AdministrationNational Healthcare Security Administration, 2020))1, urban residents’ basic medical insurance (59.70% (AdministrationNational Healthcare Security Administration, 2020)) = 2, new rural cooperative medical care (35.00% (Ma and Li, 2021)) = 3, self-financing (0.00%) = 4. Institution level:provincial level = 1, municipal level = 2, district level = 3, country level = 4. Taking logarithms of outpatient expense in the structural equation model.

We have developed a SEM to further investigate the effects of variables on outpatient expenditure (Figure 3). Variables that were unrelated to depression outpatient expenditure were excluded. Based on the fitting optimization index, the proposed SEM has a good fitting effect, χ^2^ = 4.287, df = 1,χ^2^/df = 4.287, GFI = 1.000, AGFI = 1.000, CFI = 1.000, NFI = 0.999, RFI = 0.995, IFI = 1.000, TLI = 0.996, RMSEA = 0.008. Insurance status can affect outpatient expenditure through year (β = 0.03, *p* < 0.001), age (β = −0.09, *p* < 0.001). Year (β = 0.02, *p* < 0.001) and age (β = 0.18, *p* < 0.001) can affect outpatient expenditure through institution level. However, outpatient expenditure was affected by insurance status (β = −0.02, *p* < 0.001), year (β = −0.05, *p* < 0.001), age (β = −0.02, *p* < 0.001), institution level (β = −0.31, *p* < 0.001). In addition, the year was negatively correlated with age (β = −0.04, *p* < 0.001).

## 4 Discussion

Although there are more studies on depression in China and abroad, there are few domestic studies on the economic burden of depression costs based on SHA 2011. Our study provides a comprehensive estimate of the size and distribution of the CCE of depression in outpatient clinics in Liaoning Province. Depression places a heavy financial burden on individuals, families and society. The financial burden varies widely among people with different types of depression and ages.

In terms of the financing structure, our findings show that the cost of outpatient depression treatment in Liaoning Province was mainly from publicly financed government financing programs (2015–2020: 31.97%–60.98%) and OOP payments (2015–2020: 31.88%–66.58%). Families of depressed patients face disproportionate catastrophic health expenditures and poverty due to high OOP costs (Patel et al., 2007). In India, the probability of incurring catastrophic health expenditures due to depression among women is 14.6%. Studies conducted in Pakistan and Ethiopia show that depression leads to increased healthcare costs and significant costs to families (Mogga et al., 2006; Hanlon et al., 2015). Similarly, it has also been shown that families with depression are households three times more likely to experience catastrophic OOP payments than those without depression (Liu et al., 2019). We believe that there is a reason for this high catastrophic cost; depression is a severely disabling disorder that can have persistent and recurrent distressing mood swings and somatic symptoms, and this intense distress results in seeking care from health professionals and taking medication, which inevitably leads to high costs, similar to the Ethiopian study (Mogga et al., 2006; Hanlon et al., 2015). Therefore, it is important to control the share of depression OOP in the total cost of depression and reduce the risk of poverty among residents due to medical care. If the proportion of OOP to total health costs can be reduced by less than 15% by using strategies such as progressive fee schedules, highly subsidized or free hospital services and providing certain medical services to the poor, few households will incur catastrophic expenditures.

In terms of institution type, our findings show that depression outpatient CCE is mainly concentrated in general hospitals, accounting for about 90%, with a wide variation in the proportion of Chinese hospitals, ranging from 3% to 19% and less than 1% in specialty hospitals and primary healthcare institutions; thus showing a serious imbalance in service provision in healthcare institutions. Because patients with depressive disorders exhibit multiple mood-related somatic symptoms, they tend to be seen repeatedly in various clinical departments, becoming high consumers of medical resources in healthcare institutions at all levels. Currently, in the People’s Republic of China, there are fewer resources for mental healthcare. Most Chinese patients tend to seek depression treatment at provincial and municipal general hospitals, most of which have psychiatric or psychological clinics. In Western countries, more than half of depressed patients choose to receive treatment in primary healthcare (Oxman et al., 2002), as primary care physicians with comprehensive pharmacological knowledge and psychosocial interventions are able to provide effective treatment for depression (Gensichen et al., 2006; Gilbody et al., 2006). In China, primary care is approximately 0.8 km from most urban residents, which provides support for the somatic expression of depressive tendencies in depressed patients (Jiang et al., 2018). Depression has a long treatment period and is a highly relapsing disorder, and patients require multiple follow-up visits, systematic interventions and full involvement of community general practitioners, who therefore play an important role in relapse interventions for depression (Li Qingwei, 2016). Efforts are needed to strengthen the collaboration between primary care general practitioners and mental health professionals and the identification and prevention of depression should be enhanced by integrating specialist and non-specialist multifaceted efforts.

Our findings show that the overall trend of depression outpatient CCE gradually increases during adolescence (0-19 years) and then decreases (19-24 years), to 25-59 years and then begins a fluctuating shift from a rapid decline after 60 years–69 years, and after≥70 years CCE slowly decreases and gradually convergence to zero. Depression cost differences are mainly related to the age of the patient. Some studies have shown that children are relatively less likely to suffer from depression (1%) compared to adults (Costello et al., 2003), but depression increases sharply during the elementary school years (5–11) (Kessler et al., 2012). This is consistent with significant age-related trends in the prevalence of depression that have been shown to increase gradually from the youngest to the higher ages and then decrease in the older age groups, with the prevalence consistently being lowest in the oldest age group (≥60) (Kessler et al., 2005). At the same time, it has been shown that the highest lifetime prevalence of lifelong illness is seen in adolescence with approximately 50% of lifetime illnesses being concomitant affective disorders, including depression (Guo et al., 2019). Among all cases of lifelong illness with psychiatric disorders, 50% start at age 14% and 75% at age 24 and in older stages, there is mostly co-morbidity of depression with other disorders (Kessler et al., 2005). The adolescent stage is a plastic turbulent period of life, depression or lifelong mental illness leading to suicide or disability at this stage has a huge cost in terms of personal growth, family burden, even social development. Therefore, it is very important to focus on screening for depression in adolescence and to prevent and control the harmful effects of depression.

Multiple linear regression analysis was used in this study to explore the factors influencing the cost of outpatient visits, the study factors explained 38.4% of the variance in depression outpatient costs. The findings of the multiple linear regression analysis showed that the drug standardized coefficient was 0.45, showing that patients’ choice to take medication was associated with high outpatient costs, which indicates that medication is the main modality taken by patients in the antidepressant process and that medication plays an important role in the reducing depression (Egede et al., 2016; Lekoubou et al., 2019; Torres;Granados et al., 2023). The three main drivers of depression healthcare expenditure are outpatient visits, medication, and the emergency room (Lurie et al., 2009). Continued outpatient visits and medication also increase the cost of depression. A study showed that antidepressant medication, although increasing short-term direct expenditures, significantly reduces the average medical expenditures of patients 12 months or even 5 years after depression diagnosis in the long run (Gu et al., 2020). This is important since the medication is effective and acceptable for treating depressed patients (Gill and Hatcher, 1999). At the same time, the higher cost of medication may be related to physicians’ prescribing habits, physicians’ compensation for relatively low fees for services or availability of medication to patients and the relatively low costs for patients compared to counseling (Hu, 2004). The results of our study show that, in terms of standardized coefficient B values, the impact is greater at the provincial and district levels of healthcare and less at the district level of healthcare. Also, the results of SEM showed that institution level was a significant mediating variable for the effect of year and age on the cost of depression outpatient visits. Thus, encouraging patients to go to primary care is an effective initiative to reduce the cost of outpatient visits. The standardized discourse coefficient B value of insurance status in the multiple linear regression showed that depression outpatient expenses were 0.038 and 0.027 higher than self-founded for urban workers and urban residents, indicating that medical insurance for urban workers and urban residents significantly affects depression outpatient expenses. In our SEM results, insurance status can affect outpatient expenditure through year, age. Some studies have shown that depression leads to a significant increase in commercial insurance, Medicaid, health insurance expenditures and OOP costs (Breslow et al., 2019). Depression was common among people with public insurance (Lekoubou et al., 2019). Patients with private and public insurance were prescribed more medications than those without insurance (Shao et al., 2017), patients with insurance were also more likely than uninsured patients with depression to continue antidepressant treatment for 30 days and beyond (Olfson et al., 2006). Possible reasons for these phenomena are that for patients with health insurance, medical costs are partially covered by the insurance company, which puts less financial pressure on the patient, and the patient can then be actively and sustainably treated. For patients without insurance, the high OOP costs may cause patients to abandon medication or counseling because antidepressant treatment is a very expensive treatment. Some studies have shown that people with depression have higher out-of-pocket costs on average than hypertensive patients or arthritis patients (Lurie et al., 2009), similar to those with heart disease and diabetes, which shows that the financial burden of depressed patients is quite heavy.

It is suggested that China should set up a special financial project to increase financial investment in depression, increase the number of aid patients spend on depression treatment, and increase the amount of free monthly medication subsidies for patients (Huang Fudan University, 2010). The government should improve the breadth and depth of health insurance coverage. The government should continue to reduce participation fees, especially for migrant and poor families, because many patients’ families cannot effectively use medical insurance due to the process of medical treatment in different regions in China. Since depression treatment is mainly conducted in outpatient clinics and is mostly drug-based, patients have long treatment cycles and high chronicity rates, and some patients have not recovered after 5 years (Gadit, 2004). It is therefore recommended to improve the psychotropic drug supply guarantee system, increase competition among drug manufacturers, promote the use of generic drug prescriptions, and improve the transparency of drug pricing (Lekoubou et al., 2019). By including, more antidepressant drugs in the basic medical insurance, increasing the reimbursement ratio, and realizing the free supply of some basic antidepressant drugs patients may also experience reduce financial burden due to depression. A collaborative medical-mental health governance program is formed by the integration of psychiatrists, caregivers, and psychotherapists to closely monitor the clinical outcomes of patients in treatment facilities, strengthen preventive screening and testing, and adjust treatment plans according to the patient’s illness to prevent recurrent depressive episodes and avoid the formation of intractable depression, which places a heavy burden on families and society. The treatment of depression mainly consists of prescription medication, which must be regulated by medical personnel to reduce the burden on patients. In the process of treatment, to avoid inducing demand and causing excessive medical treatment, accurate medical treatment is critical. Patients should actively cooperate with the treatment requirements, follow medical advice, take medication regularly and on time, and not stop medication without authorization. Because of the long treatment period of depression, many patients stop medication without authorization, resulting in recurrent depression, or even form refractory depression due to the seriousness of the situation.

The SHA2011-based accounting framework provides a good theoretical basis for explaining depression outpatient CCE in Liaoning Province. Under this theoretical framework, the type of institution, funding structure, and beneficiary population of depression outpatient CCE can be well explained. On this basis, we analyzed the distribution of costs for different disease types and explored the influencing factors of CCE as well as the direct and mediating effects on outpatient costs using structural equation modeling.

This analysis research has some limitations. First, Only outpatient costs for depression were reported in this study because outpatient costs accounted for more than 90% of the total costs in this study, which is due to the fact that the purchase of medications accounted for a larger portion of the outpatient costs, which is consistent with extant studies (Zhou et al., 2008). Also, patients with depression have a higher probability of visiting the outpatient clinic (Jia et al., 2003). Due to the number and percentage of hospitalization expenditure are too small, analysis of its internal composition and financing structure and influencing factors will be less accurate, which may have a certain degree of influence on the change of the trend of the cost of depression, while more than 90% of the extent of the outpatient expenditure of depression in this study can be speculated on the trend of the change of the total cost of the patient and the factors that influence it, and the comprehensive consideration of the results of the credibility of the choice to exclude the expenditure of hospitalization, but in the subsequent study we will continue to strengthen to ensure that the expenditure of the completeness of the study. Second, because depression is severely undertreated in medical institutions (Mitchell et al., 2009), it is likely that many residents of Liaoning Province who suffer from depression do not seek medical treatment, therefore some cases of depression may be underreported in the dataset. However, because the proportion of hospitalization costs is really too small, so there is no further analysis of its internal composition and financing structure and influencing factors, the proportion is too small may be less accurate analysis of the distribution of costs of various dimensions, which may have some degree of influence on the change of the trend of the cost of depression, but in the follow-up study we will continue to strengthen to ensure that the cost of the completeness of the study. Then again, because depression is mainly managed by medication or combined with counseling treatment, and patients taking medication can directly purchase online medications the data on medication may also be underreported in this dataset. Furthermore, the medical burden of disease includes direct medical expenses, indirect medical expenses, and other social losses, and this study only examined direct medical expenses. Finally, this study only considered the costs of a single depression diagnosis and did not consider the costs of comorbidities. Last but not least, the missing cases of depression were mainly from the Liaoning Provincial Hospital Specializing in Mental Diseases-Dalian No. Seven People’s Hospital, and the data from this institution with 2 years of data were deleted in this study due to missing data in some years. For these reasons, this study may underestimate the actual outpatient burden of depression in Liaoning Province.

In China, the medical burden of outpatient CCE for depressed patients is high, and outpatient pharmacotherapy is the most common treatment for depression. Therefore, it is recommended to improve the supply guarantee system of psychotropic drugs, enhance the competitiveness among pharmaceutical companies, promote the use of generic drugs medications and include more antidepressants in the essential drug list to achieve a free supply of some antidepressant essential drugs. Medical insurance is an important factor for patients to take aggressive treatment and adhere to it for a long time. Because patients with health insurance bear less out-of-pocket costs for treatment than those who are covered by health insurance, patients are more likely to actively cooperate with their doctors’ prescribed treatment or psychological counseling therapy. Therefore, it is recommended to improve the heavy burden of depression medical costs on the state, society and families by expanding the breadth and depth of health insurance coverage, consistently lowering participation fees, and increasing reimbursement rates.

## Data Availability

The original contributions presented in the study are included in the article/Supplementary material, further inquiries can be directed to the corresponding author.
